# Supplementary material for: Highly Glycolytic Immortalized Human Dermal Microvascular Endothelial Cells are Able to Grow in Glucose-Starved Conditions
Source: Biomolecules. 2019 Aug 1;9(8):332. doi: 10.3390/biom9080332 (PMC6723428; doi:10.3390/biom9080332)
Supplement: Supplementary file 1 [file biomolecules-09-00332-s001.pdf]

Supplementary Figures

Highly Glycolytic Immortalized Human Dermal Microvascular Endothelial Cells are Able to Grow in Glucose-Starved Conditions

Mª Carmen Ocaña [1,2], Beatriz Martínez-Poveda [1,2], Ana R. Quesada [1,2,3] and Miguel Ángel Medina [1,2,3,*]

[1] Universidad de Málaga, Andalucía Tech, Departamento de Biología Molecular y Bioquímica, Facultad de Ciencias, E-29071 Málaga, Spain
[2] IBIMA (Biomedical Research Institute of Málaga), E-29071 Málaga, Spain
[3] CIBER de Enfermedades Raras (CIBERER), E-29071 Málaga, Spain
[*] Correspondence: medina@uma.es; Tel.: +34 952137132

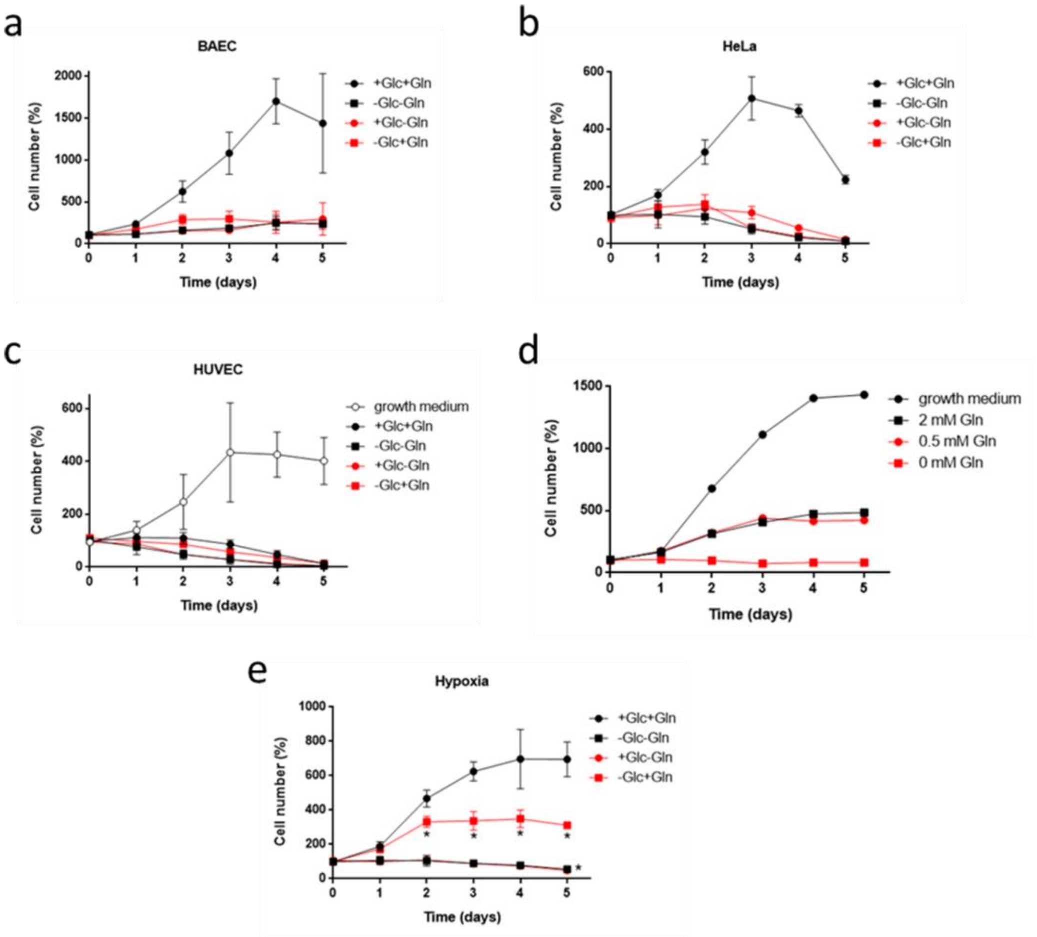

Figure S1. Cell growth in different nutritional conditions. (a) Cell growth in BAEC, (b) HeLA and (c) HUVEC was monitored in the presence or absence of 5 mM glucose and/or 0.5 mM glutamine in normoxia. Data are expressed as means ± SD of three independent experiments. $p < 0.05$ for all conditions as compared to +Glc+Gln (a–b). (d) Cell growth in HMEC was monitored in the presense of glucose and 0.5 or 2 mM glutamine. Data are expressed as the results of a unique experiment. (e) Cell growth in HMEC in the presence or absence of 5 mM glucose and/or 0.5 mM glutamine in hypoxia. Data are expressed as means ± SD of three independent experiments. *$p <$ 0.05 versus +Glc+Gln.

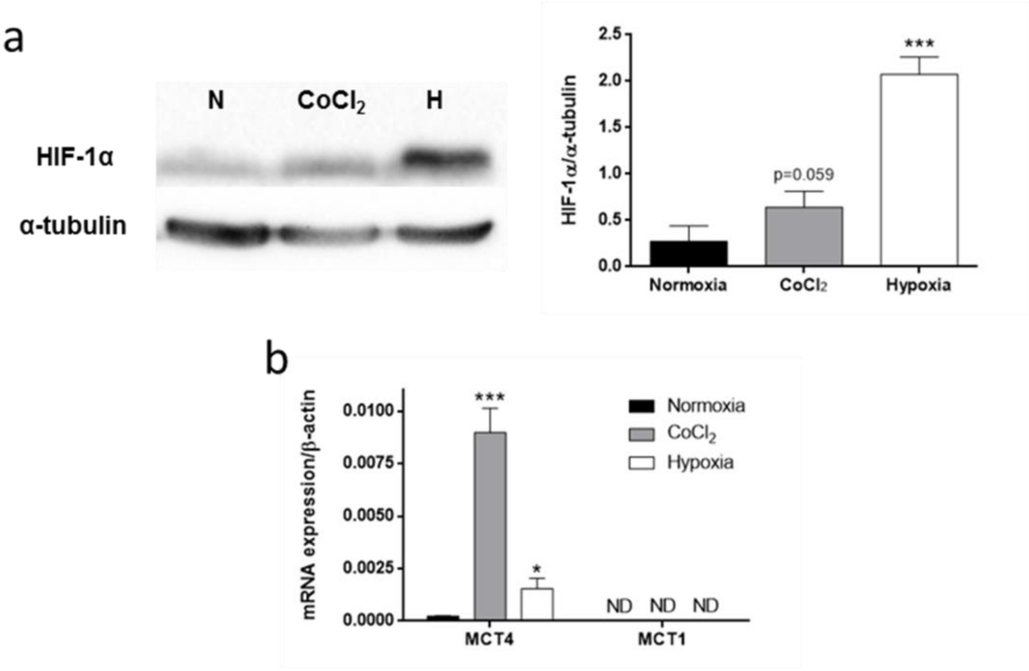

Figure 2. HIF-1α, MCT4 and MCT1 expression under hypoxic conditions. (**a**) HIF-1α protein expression was determined in HMEC under normoxia, 200 μM CoCl2 or 1% hypoxia. (**b**) MCT4 and MCT1 mRNA expression was determined in HMEC under the same conditions. Data are expressed as means ± SD of three independent experiments. *$p < 0.05$, ***$p < 0.001$ versus normoxia condition.
